# Short constrained peptides derived from phage display libraries as epitope models: the case of mAb 2F5

**DOI:** 10.1186/1742-4690-9-S2-P89

**Published:** 2012-09-13

**Authors:** Y Palacios-Rodriguez, T Gazarian, L Huerta, K Gazarian

**Affiliations:** 1 Mexican National Autonomous University, Mexico, D.F., Mexico; 2 Institute of Biomedical Research UNAM, Mexico DF, Mexico; 3 Institute of Biomedical Research, UNAM, Mexico, D.F., Mexico

## Background

Since the monoclonal antibody 2F5 (mAb 2F5) was isolated in the early 90’s, its epitope have continued to be the focus of extensive investigations attempting to elucidate the mechanism by which impedes viral entry into host cells. Because the DKW-flanking amino acids are strongly conserved in viruses, it is not clear whether the DKW only satisfies the 2F5 epitope recognition demand.

## Methods

We used phage display technology involving biopanning of a pIII-type 7-mer constrained peptide library (not screened in previous experiments with 2F5) for its epitope mimics. After peptides selection and widely characterization of several phage-peptide clones, some of them were used as immunogens. Polyclonal antibodies were evaluated as cell-cell fusion inhibitors of the CD4-Env complex interactions.

## Results

We found that the specificity of recognition of the epitope depends on the structural context in which the cognate epitope sequence is presented. The antibody does not tolerate any replacements of the DKW-flanking epitope amino acids and binds exclusively to the (L)DKWA sequence provided by a 7-mer constrained peptide exposed by the M13 phage pIII protein. Additionally, immunization data supports the notion that the binding and neutralizing immunogenic structural features of the described epitope model do not coincide.

## Conclusion

In this study, we show that when mAb 2F5 screens a pIII-type phage display 7-mer constrained peptide library for its epitope mimics, it demands an epitope sequence longer than DKW and does not tolerate substitutions in the epitope amino acid sequence as has been suggested in previous reports.

